# Dynamic preparedness metric: a paradigm shift to measure and act on preparedness

**DOI:** 10.1016/S2214-109X(22)00097-3

**Published:** 2022-04-12

**Authors:** Nirmal Kandel, Stella Chungong, WHO Technical Working Group of the Dynamic Preparedness Metric and Health Security Preparedness Department

**Affiliations:** a WHO Headquarters, Geneva 27 1211, Switzerland

Multiple indices currently exist to measure a country's preparedness for health emergencies.^1^, ^2^, ^3^, ^4^, ^5^ However, most of these metrics are based on cross-sectional assessments that do not reflect current and changing risks, particularly as related to major epidemics. Moreover, the COVID-19 pandemic has highlighted that existing measurement approaches are not sufficient to predict how a preparedness system for severe public health emergencies would perform during an emergency.^6^, ^7^ WHO aims to address these needs with the creation of a new dynamic, multi-hazards, evidence-based preparedness metric that can gauge preparedness capacity dynamically and inform key action plans for improving capacities for each country or region on the basis of identified capacity gaps.

The dynamic preparedness metric (DPM) is a composite measure with three main conceptual dimensions: hazard, vulnerability, and capacity (figure). Hazard represents the source of potential harm that a country will need to handle, based on both severity and the probability of an epidemic event or the exposure to it. Vulnerability describes the physical, social, economic, and environmental factors that could increase the susceptibility of an individual, community, asset, or system to the impact of hazards. Capacity refers to all the systems of knowledge, institutions, and infrastructure required to effectively anticipate, mitigate, respond to, and recover from the impact of a health emergency.^8^ The three dimensions are combined by informative weighting of indicators representing necessary subcomponents and elements within each dimension.

**Figure:**
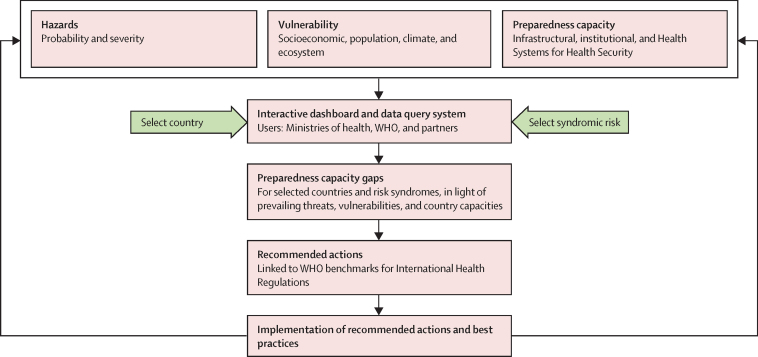
Dynamic preparedness metric workflow

Two metrics have been developed to assess the country preparedness status: (1) the risk-based DPM index, which supports countries in understanding current and changing capacities in line with hazards and vulnerabilities to strengthen their preparedness capacities for managing health emergencies effectively; and (2) the preparedness capacity gap, which helps countries identify gaps and prioritise actions necessary to address those gaps—such as those from the WHO benchmarks for International Health Regulations (IHR) capacities.

The DPM index is dynamic as it is frequently updated with publicly available data and addresses five specific disease syndromes (ie, respiratory, diarrhoeal, neurological, haemorrhagic, and acute febrile syndromes in an initial phase). The potential pathways of spread and the impact on society for each syndrome are heterogeneous; thus, the preparedness capacities and actions needed to contain each syndrome are specific. The ability of the DPM index to estimate preparedness status relative to specific syndromes and risks will better inform effective actions to increase capacities and reduce epidemic spread and its management.

Moreover, the aim of the DPM index is not to rank countries but rather to facilitate the identification of countries’ specific limitations in preparedness and possible mitigation strategies. Therefore, the DPM index is designed to support countries and regions to make evidence-based improvements in emergency preparedness considering the unique contributions of multiple sectors and disciplines. This dynamic and multisectoral approach is in line with best practices to limit the impact of future epidemics or pandemics through a health systems for health security approach.^9^ As a result, the goal of the DPM index is ultimately to inform national strategic plans, highlighting crucial areas for prioritised improvements. To achieve this goal, an online interactive dashboard has been developed for countries to access quarterly estimates of health emergency preparedness with links to suggested actions. These key actions will be informed by the goals of the WHO benchmarks for IHR (2005) capacities,^10^ which are connected to capacity scores in the DPM for each country or region. Furthermore, the DPM index can pinpoint areas where data are scarce and thus guide global health research agendas.

The WHO has finalised phase 1 of the DPM through ongoing discussion with experts in the disciplines of health security, data science, animal–human interface, infrastructure, and epidemiology, among others. The DPM index is in use for the Universal Health and Preparedness Review (UHPR) to inform the baseline preparedness status in three dimensions in addition to UHRP indicators.^11^ A working phase 1 DPM index is planned to launch in the first quarter of 2022 and implemented as a new, improved metric used in the 13th General Programme of Work (GPW 13). After phase 1, continued validation and improvement of the DPM index will be implemented to ensure that its utility will meet WHO's goals: promote health, keep the world safe, and serve the vulnerable.

I declare no competing interests. The members of the WHO Technical Working Group of the Dynamic Preparedness Metric are Woody Ang Woo Teck, Cynthia Bell, Lucy Boulanger, Garrett Brown, Orlando Cenciarelli, Matthew H Cochran, Stephane De La Rocque, Khassoum Diallo, Ande Elisha Ashasim, Kaylee Errecaborde, Sane Jussi, Julie Kae, Nirmal Kandel, Masaya Kato, Benjamin Downs Lane, Lorenzo Lionello, Rafael Lozano, Aragaw Merawi, Robert Nguni, Jennifer Nuzzo, Heather Page, Ihor Perehinets, Amit Prasad, Amelie Rioux, Dalia Samhouri, Reuben Samuel, Nahoko Shindo, Jonathan Suk, Ambrose Otau Talisuna, Schmidt Tanja, Andrew Thow, and Luca Vernaccini. The members of the Health Security Preparedness Department at WHO are Stella Chungong, Marc Ho, Qudsia Huda, Abbas Omaar, Rajesh Sreedharan, Ludy Suryantoro, Liviu Vedrasco, and Jun Xing.
